# Ocular Autoimmune Systemic Inflammatory Infectious Study (OASIS)—report 4: analysis and outcome of scleritis in an East Asian population

**DOI:** 10.1186/s12348-017-0124-5

**Published:** 2017-02-15

**Authors:** Muhammad Amir Bin Ismail, Rachel Hui Fen Lim, Helen Mi Fang, Elizabeth Poh Ying Wong, Ho Su Ling, Wee Kiak Lim, Stephen C. Teoh, Rupesh Agrawal

**Affiliations:** 1 National Healthcare Group Eye Institute, Tan Tock Seng Hospital, Singapore, 308433 Singapore; 2 Singapore National Eye Centre, Singapore, Singapore; 3 Eagle Eye Center, Mount Elizabeth Novena Hospital, Singapore, Singapore

**Keywords:** Scleritis, Epidemiology, Complications, Treatment

## Abstract

**Background:**

The purpose of this study is to evaluate the spectrum of scleritis from database of Ocular Autoimmune Systemic Inflammatory Infectious Study (OASIS) at a tertiary eye referral eye institute in Singapore. Clinical records of 120 patients with scleritis from a database of 2200 patients from Ocular Autoimmune Systemic Inflammatory Infectious Study (OASIS) were reviewed.

**Results:**

56.6% were females, with a mean age of 48.6 ± 15.9 years. 75 (62.5%) had diffuse anterior scleritis, 25 (20.8%) had nodular anterior scleritis, 7 (5.8%) had necrotizing anterior scleritis and 13 (10.8%) had posterior scleritis. Ocular complications were observed in 53.3% of patients, including anterior uveitis (42.5%), raised intraocular pressure (12.5%), and corneal involvement (11.7%). Autoimmune causes were associated with 31 (25.8%) of patients, and 10 (8.3%) patients had an associated infective etiology, much higher than Caucasian studies. 53.3% of patients were treated with oral corticosteroids while 26.7% required immunosuppressives.

**Conclusions:**

Infective etiology needs to be considered in patients of scleritis from Asian origin. In our study and in OASIS database, scleritis was associated with systemic autoimmune disease and ocular complications.

## Background

Scleritis is an uncommon potentially sight threatening ocular inflammatory disease [1, 2]. It is typically a severe painful inflammatory process characterized by inflammation of the sclera associated with engorgement of the conjunctival, superficial, and deep episcleral vessels. Based on the Watson and Hayreh classification system [3], scleritis is divided into anterior and posterior types. The anterior type is further subdivided into diffuse, nodular, and necrotizing with or without inflammation.

Ocular complications include scleral and corneal thinning, perforation, keratitis, anterior uveitis, glaucoma, cataract, and exudative retinal detachment [1, 3]. Necrotizing scleritis, in particular, has been more frequently associated with ocular complications [3, 4].

The disease may be idiopathic but previous studies have suggested that scleritis is often associated with, and may be the first sign of potentially life-threatening systemic autoimmune diseases in 30–50%. The most common are rheumatoid arthritis, granulomatosis with polyangiitis (formerly Wegener’s granulomatosis), seronegative spondyloarthropathies, relapsing polychondritis, and systemic lupus erythematosus (SLE) [5–10]. It may also be associated with infectious causes, previous ocular surgery, drugs, or malignancies [2, 6, 7].

Management of scleritis involves a multidisciplinary approach when associated with a systemic disease. Treatment is in a stepwise manner with non-steroidal anti-inflammatory drugs (NSAIDs), followed by corticosteroids, and subsequently, steroid sparing immunosuppressive agents in cases of unsatisfactory therapeutic response or if prolonged treatment is necessary. Examples of such agents are azathioprine, cyclosporine, methotrexate, cyclophosphamide, and mycophenolate mofetil (MMF) [11–13]. In cases of refractory or therapy-resistant ocular inflammatory eye disease, tumor necrosis factor alpha (TNF-α) antagonists such as infliximab and adalimumab are becoming increasingly used, together with other immunosuppressive agents [14–20]. More recently, rituximab, a CD20 monoclonal antibody, has also been described for refractory scleritis [21].

As scleritis is a relatively uncommon disorder, there are limited large studies documenting clinical experience with this disease, especially in Asian populations [22–24]. We aim to analyze our clinical experience with this disorder, in particular, the associated systemic diseases, complications, treatments methods, and outcomes of patients with scleritis presenting to a tertiary institution in Singapore from a very large database of ocular inflammatory disease—Ocular Autoimmune Systemic Inflammatory Infectious Study (OASIS) database.

## Results

One hundred and twenty patients (5.45%) with scleritis were identified during this 11-year period, out of a total of 2200 patients that presented to our Uveitis and Ocular Inflammation service. The detailed demographic and clinical characteristics of all patients included in this study are summarized in Table 1. There were 52 (43.3%) males and 68 (56.7%) females. The mean age was 48.6 ± 15.9 (range 16–83) years. Eighty three patients (69.2%) were Chinese, 15 (12.5%) Indian, 14 (11.7%) Malay, and 8 (6.7%) patients of other races. The disease was bilateral in 37 patients (30.8%). The commonest presenting complaints included eye redness (*n* = 105, 87.5%), ocular pain (*n* = 80, 66.7%), and blurring of vision (*n* = 21,17.5%).

**Table 1:** Demographic and clinical characteristics of patients with scleritis from Ocular Autoimmune Systemic Inflammatory Infectious Study (OASIS) database

Characteristics	No. of patients, *n* (%)
Mean age ± SD (years)	48.6 ± 15.9
Range	16–83
Gender	
Male	52 (43.3%)
Female	68 (56.7%)
Bilaterality	37 (30.8%)
Race	
Chinese	83 (69.2%)
Indian	15 (11.7%)
Malay	14 (11.7%)
Others	8 (6.7%)
Ocular complications (any)	64 (53.3%)
Anterior segment	
Anterior uveitis	51 (42.5%)
Corneal involvement (ulceration, keratitis)	14 (11.7%)
Ocular hypertension	15 (12.5%)
Posterior segment	
Cystoid macular edema	3 (2.5%)
Exudative retinal detachment	5 (4.2%)
Optic disc swelling	3 (2.5%)
Systemic associations	
Autoimmune	31 (25.8%)
Rheumatoid arthritis	9 (7.5%)
Granulomatosis with polyangitis	5 (4.2%)
Relapsing polychondritis	4 (3.3%)
Sjogren’s syndrome	4 (3.3%)
Psoriatic arthropathy	2 (1.7%)
Systemic lupus erythematosus	1 (0.8%)
Behcet’s disease	1 (0.8%)
Churg-Strauss syndrome	1 (0.8%)
Ulcerative colitis	1 (0.8%)
Takayasu’s arteritis	1 (0.8%)
Non-specific autoimmune disease	1 (0.8%)
Orbital inflammatory syndrome	1 (0.8%)
Other non-autoimmune systemic:	2 (1.7%)
Myelodysplastic syndrome	1 (0.8%)
Polyclonal gammopathy	1 (0.8%)
Presumed infectious association	10 (8.3%)
TB T-spot positive	5 (4.2%)
Herpes zoster ophthalmicus	2 (1.7%)
Herpes simplex viral keratitis	1 (0.8%)
Acquired immunodeficiency syndrome	1 (0.8%)
Hansen’s disease	1 (0.8%)
Recurrence	20 (16.7%)
Treatment	
Topical steroids	114 (95.0%)
Oral non-steroidal anti-inflammatory drugs	63 (47.3%)
Oral corticosteroids	64 (53.3%)
Systemic immunosuppressant (any)	32 (26.7%)
Azathioprine	16 (13.3%)
Methotrexate	11 (9.2%)
Mycophenolate mofetil	6 (5.0%)
Sulphasalazine	6 (5.0%)
Cyclosporine	4 (3.3%)
Cyclophosphamide	4 (3.3%)
Tumor necrosis factor-α antagonist (infliximab)	2 (1.7%)

Diffuse anterior scleritis (*n* = 75, 62.5%) was the most common type of scleritis, followed by nodular (*n* = 25, 20.8%), posterior (*n* = 13, 10.8%) and necrotizing anterior scleritis (*n* = 7, 5.8%). Demographic and clinical characteristics of the subtypes of scleritis are summarized in Table 2. The mean age at presentation was highest for necrotizing anterior scleritis (63.7 ± 16.5 years) compared to other forms of scleritis (48.6 ± 14.1 years for diffuse, 44.0 ± 16.9 for nodular, and 49.2 ± 20.3 for posterior). There were statistically significant differences in age among the 4 subtypes of scleritis (*p* = 0.037). Post hoc pairwise comparisons showed that patients in total necrotizing subtype were 19.7 years older than patients in nodular subtype (95% confidence interval (CI) from 1.8 to 37.5, adjusted *p* = 0.022).

**Table 2:** Demographic and clinical characteristics of patients classified into subtypes of scleritis (no. of patients, *n* (%))

	Diffuse	Nodular	Necrotizing	Posterior
Number of patients	75 (62.5)	25 (20.8)	7 (5.8)	13 (10.8)
Age (mean) years	48.6	44.0	63.7	49.2
Range	20–74	18–79	38–83	16–80
Gender				
Male	32 (42.7)	12 (48.0)	2 (28.6)	5 (38.5)
Female	43 (57.3)	13 (52.0)	5 (71.4)	8 (61.5)
Ocular complications *(*any*)*	37 (49.3)	10 (40.0)	6 (85.7)	12 (84.6)
Anterior segment				
Corneal involvement (ulceration, keratitis)	7 (9.3)	4 (16.0)	2 (28.6)	1 (7.7)
Ocular hypertension	9 (12.0)	2 (8.0)	2 (28.6)	2 (15.4)
Anterior uveitis	30 (40.0)	8 (32.0)	5 (71.4)	8 (61.5)
Posterior segment				
Vitritis	0	0	0	3 (23.1)
Cystoid macular edema	0	0	0	3 (23.1)
Exudative retinal detachment	0	0	1 (14.3)	4 (30.8)
Optic disc swelling	1 (1.3)	0	0	2 (15.4)

### Complications

At least one ocular complication was observed in 53.3% of all patients either at presentation or at follow up. Corneal involvement (ulceration, keratitis), high intraocular pressure, and anterior uveitis constituted major anterior segment complications while vitritis, cystoid macular edema, exudative retinal detachment, and optic disc swelling were the major posterior segment complications. Ocular complications were present in 49.3% (*n* = 37) of diffuse, 40.0% (*n* = 10) of nodular, 85.7% (*n* = 6) of necrotizing, and 84.6% (*n* = 11) of posterior scleritis. Posterior scleritis and necrotizing scleritis were associated with higher rates of ocular complications (*p = 0.014*). The most common complication was anterior uveitis, which occurred in 51 (42.5%) of patients.

Posterior segment complications were most commonly observed in patients with posterior scleritis *(p < 0.001*). This included exudative retinal detachment (RD) (30.8%, *n* = 4), cystoid macular edema (23.1%, *n* = 3), and optic disc swelling (15.4%, *n* = 2).

### Systemic associations

Associated autoimmune and infective associations are summarized in Table 3. Autoimmune and infective causes were associated with 31 (25.8%) and 10 (8.3%) of scleritis patients, respectively. The most common associated autoimmune cause was rheumatoid arthritis (7.5%), followed by granulomatosis with polyangiitis (4.2%), relapsing polychondritis (3.3%), Sjogren’s syndrome (3.3%), and psoriatic arthropathy (1.7%). Necrotizing anterior scleritis had the highest proportion with an autoimmune condition (85.7%), followed by posterior scleritis (30.8%), and diffuse scleritis (25.3%), *p* = 0.001. Only 2 out of 25 (8.0%) patients with nodular scleritis patients had an associated autoimmune conditions.

**Table 3:** Associated autoimmune and systemic disease (no. of patients, *n* (%))

	Diffuse	Nodular	Necrotizing	Posterior
Number of patients	75 (62.5)	25 (20.8)	7 (5.8)	13 (10.8)
Systemic autoimmune disease	19 (25.3)	2 (8.0)	6 (85.7)	4 (30.8)
Rheumatoid arthritis	6 (5.0)	0	3 (42.9)	0
Granulomatosis with polyangitis	3 (4.0)	0	0	2 (15.4)
Relapsing polychondritis	2 (2.7)	1 (4.0)	1 (14.3)	0
Psoriatic arthropathy	2 (2.7)	0	0	0
Presumed infectious association	4 (5.3)	6 (24.0)	0 (0)	0 (0)

Infective screen was done in 41 patients. The most commonly associated infective cause was positive test for latent mycobacterial infection. Ten showed a positive TB T spot, and 3 were indeterminate. A disproportionately high percentage of nodular scleritis (6 patients out of 25, or 24%) were associated with a possible infective cause, while only 8% had an associated autoimmune cause. Nodular scleritis was found to have a higher association with a probable infective etiology.

### Treatment

Treatment modalities for all patients are shown in Table 4. One hundred and fourteen (95%) were treated with topical corticosteroids as first-line treatment. Sixty-three patients (52.5%) were treated oral NSAIDs, 64 (53.3%) patients were treated with oral corticosteroids, and 26.7% needed immunosuppressants including azathioprine (*n* = 16, 13.3%), methotrexate (*n* = 11, 9.2%), MMF (*n* = 6, 5%), sulphasalazine (*n* = 6, 5%), hydroxychloroquine (*n* = 5, 4.2%), cyclophosphamide (*n* = 4, 3.3%), and cyclosporine (*n* = 4, 3.3%). Intravenous methylprednisolone was given to 5 patients (4.2%), and 2 patients required biologic agents (intravenous Infliximab) (1.7%). The choices of oral NSAIDs or systemic steroids/immunosuppressants were made clinically, depending on the severity of the scleritis.

**Table 4:** Treatment and recurrence (no. of patients, *n* (%))

	Diffuse	Nodular	Necrotizing	Posterior
Number of patients	75 (62.5)	25 (20.8)	7 (5.8)	13 (10.8)
Topical steroids	71 (94.7)	25 (100)	6 (85.7)	12 (92.3)
Oral NSAID	40 (53.3)	16 (64.0)	3 (42.9)	4 (30.8)
Oral corticosteroids	37 (49.3)	9 (36.0)	6 (85.7)	12 (92.3)
Oral immunosuppressives (excluding prednisolone)	19 (25.3)	3 (12.0)	5 (71.4)	5 (38.5)
Oral steroid or immunosuppressives	40 (53.3)	9 (36.0)	6 (85.7)	12 (92.3)
Recurrence	14 (18.7)	2 (8.0)	2 (28.6)	2 (15.4)

Among the 100 patients with non-necrotizing (diffuse and nodular) anterior scleritis, 56 (56.0%) received oral NSAIDs as first-line therapy, with 96 patients (96.0%) treated with topical steroids. Out of the 56 patients receiving NSAID therapy, 41 (41.0%) responded and required no further treatment. Fifteen patients (15.0%) were unresponsive to therapy and were given either systemic corticosteroids alone or in combination with immunosuppressive drugs. For 31 patients (31.0%), the first choice treatment was systemic corticosteroids.

Oral corticosteroids were used in 85.7% of necrotizing anterior scleritis and 92.3% of posterior scleritis, compared to 49.3% of diffuse scleritis and 32% of nodular scleritis. Around 70% of necrotizing anterior scleritis and 38.5% of posterior scleritis patients required immunosuppressive agents while only 24.0% and 12.0% of diffuse and nodular scleritis required immunosuppressive treatment.

Anti-tubercular therapy for 6 months was given in the patients with latent tubercular disease.

### Recurrence and resolution

Recurrence of scleritis occurred in 20 patients (16.7%), with the largest proportion of recurrence amongst patients with necrotizing anterior scleritis (*n* = 2, 26.6%), followed by diffuse anterior scleritis (*n* = 14, 18.7%) (*p* = 0.463).

Resolution of inflammation was longer amongst patients with necrotizing sclerosis, and occurred in 19.3 days in diffuse, 21.2 days in nodular. and 75 days in necrotizing scleritis.

## Discussion

Scleritis is an uncommon inflammatory disease that may cause complications if not treated in a timely manner. This retrospective study involved 120 patients with scleritis seen over an 11-year period in a tertiary eye center in Singapore.

The mean age of our patients were 48.6, slightly younger compared to the Caucasian population (51.5 years) [9]. Our female to male ratio was 1.3:1, similar to the rates reported in the literature (1.27:1 and 1.57:1) [4, 9, 27]. Around 31% of patients had bilateral disease, similar to the data reported by Sainz de la Maza et al. (34%) [4].

Laboratory evaluation revealed positive autoimmune markers in 34 patients (28.3%). Fourteen (11.7%) were RF positive, 13 (10.8%) were ANA positive, 9 (7.5%) were ANCA positive, and 3 (2.5%) were anti-dsDNA (DsDNA) positive. Out of these, three were newly diagnosed with RA, three granulomatosis with polyangiitis, and one with SLE. Our results showed that laboratory investigations are useful in diagnosing an underlying systemic disease associated with scleritis. Screening for an underlying systemic disease should be aimed at diseases with higher associations such as RA and granulomatosis with polyangiitis. As presence of scleritis is associated with a worse prognosis and a higher mortality rate in patients with rheumatoid arthritis, initial systemic evaluation and close follow-up of patients with idiopathic scleritis and rheumatoid factor positive is crucial [25].

Previous series from North America and Europe have demonstrated that systemic autoimmune diseases are present in approximately 30–50% of patients with scleritis [3, 4, 26]^*.*^ In our study, 25.8% had a systemic rheumatologic disease. This is similar to other Asian studies which showed an association in 15–22% of patients [24, 27–29]. Our findings similarly suggest that the association with systemic diseases might be lower in Asian populations with scleritis compared to North American and European populations [30, 31]. The most common rheumatologic disease is RA, followed by granulomatosis with polyangiitis, relapsing polychondritis, Sjogren’s syndrome, and psoriatic arthropathy. A comparison of the results from our study with previously published data (other Asian, European, and North American studies) are summarized in Table 5.

**Table 5:** Comparison of the results from our series with previously published data (%)

	Our study	Other Asian Studies [24, 29–31]	European studies [32, 33]	North American studies [27]
Associated autoimmune disease	25.8	15–22	32.7–52	35.8–39
Infectious cause (TB)	4.2	7.2	0	0–2
Systemic steroid therapy	53.3	42.2	61.5	31.9–36.7
Immunosuppressive therapy	26.7	22.9	34–36	26.1–38.0

Associated infective screen was positive in 10 patients (8.3%), with TB being the most common in our study. Three of the patients with presumed TB-related nodular scleritis were foreign nationals working in Singapore (2 from China, and 1 from India).

Based on current literature, an infectious cause is found in 4–18% of patients, of which herpes zoster is the most common, followed by TB, syphilis, leprosy, and Lyme borreliosis [3, 6, 9, 32]. A study by Tabara [33] has suggested that TB might be involved in the pathogenesis of scleritis in some patients, particularly in eyes that present with localized elevated nodules of the sclera. Asian patients with nodular scleritis may benefit from screening for an infective agent such as TB, as they will require a different therapeutic approach. It is also important to note that TB is still an important infective cause in the Asian patient who presents with scleritis.

In addition to autoimmune diseases and infections, it is also important to bear in mind that a malignancy can be the underlying disease in a patient with scleritis. Posterior scleritis has been reported in patients with lymphoma, multiple myeloma, and Waldenstrom macroglobulinemia [34]. In our study, there was one patient with myelodysplastic syndrome and another with polyclonal gammopathy. Medication, surgery, and trauma are reported to be rare in all studies.

Management of non-necrotizing anterior scleritis consists of NSAIDs in combination with topical corticosteroids as first line based on published data [2, 5, 35, 36]. 21% of patients with non-necrotizing anterior scleritis required additional immunosuppressive agents while 85.7% necrotizing, and 30.8% of posterior scleritis required immunosuppressive therapy. In the study by Jabs et al. [8], nearly 67% of patients with scleritis required more than NSAID therapy, and 26% needed immunosuppressant therapy. In our study, we found a similar treatment pattern with 65.8% of patients needing more than NSAIDs to control the inflammation, and 25.8% were treated with immunosuppressive drugs.

TNF-α (infliximab) is a biologic immunomodulatory agent used as first line for Behcet’s disease or for ocular complications of rheumatoid arthritis when patients have failed combination treatment with one or more immunosuppressive agents [35] In our series, 2 patients (1.7%) were started on infliximab treatment. One was due to necrotizing anterior scleritis secondary to relapsing polychondritis, and another due to nodular scleritis secondary to rheumatoid arthritis. Both failed treatment with corticosteroids, azathioprine, cyclosporine, and mycophenolate mofetil. In general, for this study, intravenous infliximab were used only after failure of several different types of immunosuppressive agents in a step ladder increment, due to financial factors as insurance does not cover the costs of these new agents locally.

Resolution of inflammation occurred in 19.3 days in diffuse, 21.2 days in nodular, and 75 days in necrotizing scleritis. In the study by Tuft and Watson [9], patients with diffuse scleritis received treatment for the shortest period, whereas the mean duration of treatment was longest for the necrotizing group. This finding is consistent with our study which showed that resolution of inflammation is shortest in diffuse and longest in necrotizing scleritis. Recurrence rate is highest among the necrotizing scleritis, with 28.6% of patients having at least 1 recurrence.

The limitations of our study lie mainly in the retrospective nature, with a relatively small sample size collected from a single institution. This may not fully reflect the spectrum of scleritis of our entire local population. Furthermore, the treatment modalities may differ depending on the preferences of the ocular inflammation specialists. Nonetheless, this is a relatively uncommon condition, and our data was obtained over a long 11-year period. The results of our study add to the knowledge of the epidemiology of scleritis in an Asian clinical setting.

## Conclusions

In conclusion, scleritis is a severe ocular inflammation which may be idiopathic, or associated with autoimmune or infectious cause. While infectious causes are uncommon, Asian patients with nodular scleritis may need screening for an infective agent such as TB as this may alter the management of this disease. Treatment may involve a step ladder management strategy with oral NSAIDs, oral corticosteroids, and immunosuppressants/biologic agents. Close monitoring of complications, especially for necrotizing scleritis and posterior scleritis, systemic evaluation, and timely treatment are necessary for these patients.

## Methods

We retrospectively analyzed the case records of all consecutive new scleritis cases diagnosed at the Uveitis and Ocular Inflammation service of our hospital, from January 2004 to December 2014 from 2200 patients in the OASIS database.

Data collected included information such as age at diagnosis, race, gender, anatomic location of inflammation, presenting complaints, ocular complications, associated systemic or inflammatory disease, investigations, use of topical and/or systemic treatments, and recurrence.

The diagnosis of scleritis was based on a comprehensive clinical history including a systemic review, detailed ocular examination, and investigations when deemed necessary. The Watson system was used to classify the type of scleritis [3], with the diagnosis of posterior scleritis confirmed by ultrasonography and/or CT of the orbit [34]. In all the cases, the diagnosis of scleritis was made by a uveitis specialist in our ocular inflammation service.

Complications were divided into anterior and posterior segment complications. Corneal complications included ulcerative or peripheral thinning, while anterior uveitis was diagnosed when 1+ cells (based on the Standardization of Uveitis Nomenclature Working Group Criteria) [37] or more were observed in the anterior chamber. Ocular hypertension was defined as an intraocular pressure of more than 21 mmHg measured by the Goldmann applanation tonometer (GAT). The presence of cystoid macular edema was noted clinically and confirmed by further investigations such as optical coherence tomography or fundus fluorescein angiography. Serous retinal detachment was diagnosed on fundoscopy or ultrasound.

Laboratory testing included blood tests, urine tests, and a chest radiograph. Blood tests included a complete blood count (CBC) and white cell differential, acute phase reactants such as erythrocyte sedimentation rate (ESR) and C-reactive protein (CRP), liver and renal function tests. Autoimmune markers such as rheumatoid factor (RF), anti-nuclear antibodies (ANA), anti-double-stranded DNA (anti-dsDNA), anti-neutrophil cytoplasmic antibody (ANCA) were also included. Other tests, which include infective screening for tuberculosis (TB) using the tuberculosis interferon gamma release assay (T-SPOT.TB, Oxford Immunotec, UK), treponemal serology (syphilis IgG and Venereal Disease Research Laboratory (VDRL)), were not taken routinely, but when deemed necessary based on history and physical examination. A history of scleritis with discharge, or associated trauma, and examination findings of a yellowish nodular lesion was deemed more suspicious for an infective cause and further investigations were performed.

Associated systemic diseases were classified into infectious or autoimmune. These were diagnosed based on the results of a compatible history, clinical features, and laboratory data, often in conjunction with rheumatologists and infectious disease physicians.

Treatment involved oral NSAIDs, topical, and/or systemic corticosteroids depending on the severity, location, and chronicity of the inflammatory process, as well as the risk of visual loss and complications. Immunosuppressive drugs were used as a second-line therapy when there was failure of response to oral corticosteroid monotherapy, development of intolerable side effects to corticosteroids, or in the presence of associated systemic rheumatological disease.

Recurrence was defined as repeated episodes of scleritis separated by periods of inactivity over 3 months in duration without treatment. The duration of follow-up was also obtained.

All data were entered into a computerized database system for descriptive analysis and analyzed with IBM SPSS Statistics software (version 22, IBM, New York, USA) and R (version 3.1.2, The R Foundation for Statistical Computing), with continuous variables expressed as mean with standard deviation, and range and categorical variables as percentages. Pearson’s chi-square test was performed to analyze association between the groups of patients and their demographics and clinical characteristics. If expected counts within a category were less than 5 for more than 20% of cells, Fisher’s exact test was used instead. Age among the groups of patients were compared using one way analysis of variance (ANOVA), and post hoc pairwise comparisons done with Bonferroni correction applied if the ANOVA showed results of statistical significance. A *p* value of <0.05 was considered to indicate statistical significance.

The study was approved by the National Healthcare Group Domain Specific Review Board, the conduct of which is overseen by the National Healthcare Group Research Ethics Committee.
